# Transmission efficiency of *Cotton leaf curl Multan virus* by three cryptic species of *Bemisia tabaci* complex in cotton cultivars

**DOI:** 10.7717/peerj.7788

**Published:** 2019-10-01

**Authors:** Ting Chen, Qamar Saeed, Zifu He, Lihua Lu

**Affiliations:** 1 Plant Protection Research Institute, Guangdong Academy of Agricultural Sciences, Guangzhou, Guangdong, China; 2 Guangdong Provincial Key Laboratory of High Technology for Plant Protection, Guangzhou, China; 3 Entomology, Department of Entomology, Bahauddin, Zakariya University, Multan, Pakistan

**Keywords:** *Bemisia tabaci*, Transmission, Cryptic species, *Cotton leaf curl Multan virus*, *Gossypium hirsutum*

## Abstract

*Cotton leaf curl Multan virus* (CLCuMuV) is a serious and economically important viral disease agent in cotton and ornamental plants like Hibiscus in many regions of the world, especially in South Asia. CLCuMuV is transmitted exclusively by *Bemisia tabaci* cryptic species complex. This virus was recently recorded in southern China, presumably an invasion from South Asia. This study was performed to estimate the efficiency of three species of the* B. tabaci* whitefly complex (tentatively named as MEAM1, MED and Asia II 7, respectively) to transmit CLCuMuV and Cotton leaf curl multan virus betasatelite (CLCuMuB). Transmission assays and real-time quantitative PCR were conducted using three cultivars of cotton, *Gossypium hirsutum*, including 112-2, Xinhai-21 and Zhongmian-40. The results indicated that Asia II 7 was able to transmit the virus to two of the cotton cultivars, i.e. 112-2 and Xinhai-21, with the highest transmission efficiencies of 40% and 30%, respectively, but was unable to transmit the virus to the cotton cultivar Zhongmian-40. MEAM1 and MED failed to transmit CLCuMuV and CLCuMuB to any of the three cotton cultivars. After the three cryptic species of whiteflies had fed on virus-infected cotton plants for 48 h, the relative quantity of CLCuMuV in Asia II 7 was detected to be significantly higher than that in both MEAM1 and MED (*P* < 0.05). These results indicate that among the three species of whiteflies Asia II 7 is likely the most efficient vector for CLCuMuV and CLCuMuB in *Malvaceae* crops in China. Our findings provide valuable information to the control of viral diseases caused by CLCuMuV in the field.

## Introduction

The sweet potato whitefly, *Bemisia tabaci* (Gennadius) (Hemiptera: Aleyrodidae), causes significant damage in different agricultural regions, not only by direct feeding as a pest but also by its ability to efficiently transmit several *Geminiviruses*, and is responsible for a number of epidemics worldwide (Gilbertson et al., 2015). The whitefly taxonomic group is believed to be a complex of over 39 cryptic species (Liu, Colvin & De Barro, 2012; Boykin & De Barro, 2014; Alemandri et al., 2015). Different cryptic whitefly species may transmit specific begomoviruses with various levels of efficiency, and each of the whitefly species may only be able to transmit certain viruses (Bedford et al., 1994; Polston, Barro & Boykin, 2014). Cotton leaf curl disease (CLCuD) is one of the most significant diseases in Pakistan and India (Briddon & Markham, 2000; Briddon et al., 2003; Sattar et al., 2013), the whitefly vectors of begomoviruses associated with the development of this disease. Field survey of whitefly vectors have been conducted in the area of CLCuD-epidemic field. Asia II 1 is the dominant cryptic species of the whiteflies in Pakistan (Ahmed et al., 2011; Ashfaq et al., 2014; Masood et al., 2017; Islam et al., 2018). Asia I, Asia II 5, Asia II 7 and Asia II 8 are identified in the field of cotton in India (Dinsdale et al., 2010; Rajagopalan et al., 2012; Ellango et al., 2015). In field surveys, it was observed that plants exhibiting symptoms of CLCuD in Guangdong Province, China, were also infested with the whitefly populations comprising of the MEAM1 and Asia II 7 cryptic species (Chen et al., 2016a). There are 11 viruses that have been identified to be associated with the leaf curl disease of cotton (Pan et al., 2018). However, whitefly transmission of viruses in cotton and Hibiscus was experimentally determined without stating the species of virus and whitefly used (Singh, 2001; Khan & Ahmad, 2005; Rajeshwari et al., 2005).

*Cotton leaf curl Multan virus* (CLCuMuV), associated with a betasatellite called Cotton leaf curl Multan betasatellite (CLCuMuB), was the major pathogen to cause CLCuD in Multan, Pakistan, in the 1990s (Briddon & Markham, 2000; Mansoor et al., 2003). The virus rapidly spread to all cotton-growing areas of Pakistan, Northwestern India (Sattar et al., 2013). Thus far, CLCuMuV has been found in Pakistan, India and the Philippines (Briddon et al., 2003; Srivastava et al., 2016; She et al., 2017). In China, CLCuMuV spread rapidly in the last thirteen years and became established in Southern China. It infects at least five malvaceous plant species, *H. rosa-sinensis*, *H. esculentus*, *Malvaviscus arboreus*, *Gossypium hirsutum* and *H. cannabinus* (Cai et al., 2010; Du et al., 2015)*.* Experimental inoculation demonstrated that CLCuMuV and CLCuMuB infectious clones can cause typical symptoms of the disease on cotton and several other *Malvaceae* plants. The symptoms include upward or downward curling of the leaf margins, vein thickening and enation (Tang et al., 2015).

Much more attention has been paid to the whitefly species associated with the CLCuMuV in recent years. Previous studies in the laboratory showed that CLCuMuV and CLCuMuB was transmissible through indigenous Asia II 7 and Asia II 1 species and caused CLCuD symptoms in kenaf and okra plants (Chen et al., 2016b). Lately, tobacco plant was used as a virus source; and the indigenous Asia II 1 species was able to transmit disease-causing CLCuMuV to cotton and tobacco (Pan et al., 2018). Field surveys conducted on *Malvaceae* hosts showed that Asia II 7 was the dominant indigenous cryptic species of whitefly in South China. *Hibiscus* serves as an ornamental flower and is transported from southern China to flower markets in other provinces in China. Perhaps via international trade in ornamental plants, this plant serves as a continuous source of begomoviruses. Therefore, an evaluation of the potential of whitefly species to cotton plants will help prevent the spread of the virus.

MEAM1and MED are the most abundant whiteflies across china and in many other countries (De Barro et al., 2010). In this study, we conducted laboratory experiments to compare the transmission and acquisition ability of CLCuMuV by the invasive cryptic species MEAM1, MED, and the indigenous Asia II 7 *B. tabaci* complex in the *Gossypium hirsutum*. The data can contribute to broader knowledge on whitefly begomovirus transmission and can help us in devising effective control strategies against CLCuD.

## Materials & Methods

Whitefly insects belonging to three cryptic species (MEAM1, MED and Asia II 7) were collected from three localities in Guangdong Province, China, and were maintained in the Guangdong Provincial Key Laboratory of High Technology for Plant Protection, Plant Protection Research Institute, Guangdong Academy of Agricultural Sciences, Guangzhou (Table 1). Pure populations of three whitefly species were maintained on cotton, *G. hirsutum* cv. Zhongmian-838, which is a suitable host for all three species and a non host of CLCuMuV (Tang et al., 2017). The whiteflies rearing were conducted in insect proof cages (60 cm × 60 cm × 60 cm) in a climate-controlled room at 26 ± 1 °C, 16/8 h light/dark, and 50–70% relative humidity. The purity of each culture was tested on a monthly basis by a random sampling of 30 adults as described by Chen et al. (2016b) using the mitochondrial cytochrome oxidase I (*mtCOI*) PCR technique.

**Table 1:** Three species of the whitefly *Bemisia tabaci* complex collected from Guangdong Province, China.

**Cryptic species**	**Locality of collection**	**Date ofcollection**	**Geographic location**	**Plant**	**mtCOI Genbank access no.**
Asia II 7	Qingyuan, Guangdong	2014-11	23°44′37″N, 113°27′14″E	Hibiscus	KM821541
MEAM1	Guangzhou, Guangdong	2014-8	23°9′28″N, 113°22′9″E	Eggplant	KM821540
MED	Heyuan, Guangdong	2015-8	23°36′34″N, 114°36′09″E	Tomato	GQ371165

### Virus source for inoculation

An infectious clone of CLCuMuV was used to inoculate cotton plants (*G. hirsutum* cv. 112-2), CLCuMuV (accession number KP762786) and CLCuMuB (KP762787) was isolated from diseased cotton in the Guangdong province of China. The plants were agroinoculated with CLCuMuV and CLCuMuB infectious clone pGreenII049-1.6A and pGreenII049-2.0β at the 2-3-true-leaf stage (Wang et al., 2016). At 20 days post-inoculation, the success of virus infection of source cotton plants was assessed by the appearance of leaf curling and vein thickening. The diseased plants were maintained in an insect-free room at 26 ± 1 ° C with a 16 h and 8 h of light and dark period respectively, for transmission tests.

### Experimental plants

The cotton plants *G. hirsutum* cv. 112-2, Xinhai-21, and Zhongmian-40 were planted in plastic pots (15 × 15 cm). The plants were grown and maintained in separate cages following standard procedures for growing cotton crops. All plants were grown in an insect-free greenhouse with a controlled temperature of 24–28 °C and 16 h and 8 h of light and dark period respectively. Cotton plants were raised to the 3–5-true-leaf stage in pots for transmission tests.

### Transmission of CLCuMuV and CLCuMuB to cotton plants by whiteflies

For each of three whitefly species, approximately 7-8 days old, 500 whitefly adults were collected and placed on the CLCuMuV and CLCuMuB infected-cotton plants for 48 h of acquisition access period (AAP) to acquire the virus. 10 viruliferous adults were collected on the infested plants and caged on test plants to feed for another 48 h of inoculation access period (IAP). Plastic gauze cages (12 cm  ×12 cm) (homemade for insect-proof) were used to enclose the whiteflies on the top leaves of test plants. Inoculated plants were sprayed with imidacloprid (60 mg/L) to kill all the adults and eggs and kept for symptom development in insect-proof cages. Each treatment had three replicates with each consisting of 20 plants. The plants in each treatment were observed daily for the development of disease symptoms. After 90 days, the cotton plants in each of the treatments were determined by inspection for symptoms and PCR.

### Detection of CLCuMuV and CLCuMuB DNA in diseased cotton plants

Nucleic acids from cotton plants were extracted using the method of Kidwell & Osborn (1992) with slight modifications (primer sequences used for PCR analysis are shown in Table 2). PCR was conducted with 25 micro liter volumes containing 12.5 µL PCR Mixer (Takara Bio, Mountain View, CA, USA), two µL template DNA lysate (∼20 ng), one µM of each primer, 0.25 µM of each deoxynucleoside triphosphate, and 8.5 µL sterile water. Amplifications were performed in a DNA Engine PTC-200 Thermal Cycler (Bio-Rad, Hercules, CA, USA). The PCR was set to an amplification cycle consisting of an initial denaturing at 94 °C for 4 min, followed by 35 cycles of 94 °C for 45 s, 52 °C for 45 s and 52 °C for 60 s and a final extension of 72 °C for 10 min. PCR products of a volume of 10 micro liters were electrophoresed on a 1.0% agarosgel in a 1 × TAE buffer and visualized by a Universal Hood II system (Bio-Rad, Hercules, CA, USA).

**Table 2:** Primer sequences used for PCR analysis.

**Gene name**	**Primer name**	**Primer sequence**	**Amplicon size**	**References**
CLCuMuV	CL-F	CAGGAAGCAGGAAAATACGAGA	831	Tang et al. (2015)
	CL-R	TGGCAGTCCAACACAAAATACG		
CLCuMuB	beta-F	AAGTCGAATGGAACGTGAATGT	837	Tang et al. (2015)
	beta-R	GGAGACCAAAAGAGGAGAGAGA		
CLCuMuV	CLq-F	ACACTTGTGCAGTCCCAGAG	97	present study
	CLq-R	CACTTCAACCGTCCATTCAC		
CLCuMuB	betaq-F	CCCCGTTGTATGCGAATAGGAAA	133	present study
	betaq-R	TCCACCAAGTCACCATCGCTCAT		
β-actin	β-actin-F	TCTTCCAGCCATCCTTCTTG	130	Guo et al. (2015)
	β-actin-R	CGGTGATTTCCTTCTGCATT		

### Detection of CLCuMuV and CLCuMuB DNA by qPCR in whitefly adults

Quantification of CLCuMuV and CLCuMuB in whitefly individuals was performed after various feeding durations on CLCuMuV and CLCuMuB -infected cotton plants. Five hundred nonviruliferous 7 to 8-day-old adults of each species were transferred to feed on CLCuMuV and CLCuMuB -infected cotton plants to acquire the virus. Two detection experiments were conducted on the whiteflies. In the first experiment, 10 female and 10 male adults were collected after a 48 h AAP. In the second experiment, 10 adults without the discrimination of sex were randomly collected from each plant at the end of a 6, 12, 24 and 48 h AAP, respectively. Each experiment was replicated three times. The collected whitefly samples were stored at −20 °C.

The DNA of the 10 whitefly adults was extracted with an Easy Pure Genomic DNA Kit (Trans Gen Biotech, Beijing, China). The DNA samples were stored at −20 °C. The primer sequences used for real-time PCR analysis are shown in Table 2. The β-actin gene was used in each sample as a normalization gene to verify equal quantity of whitefly genomic DNA. Amplifications were performed using SYBR^®^ Premix Ex Taq™ (Takara, Liaoning, China) and a CFX 96™ real-time PCR system (Bio-Rad, Hercules, CA, USA) with SYBR green detection (Takara, Liaoning, China). The relative quantity of viral DNA was calculated using the 2−ΔCt method (Guo et al., 2015).

### Statistical analyses

The relative quantity of CLCuMuV and CLCuMuB in ten adult whiteflies were used for the transmission tests and were compared by one-way analysis of variance (ANOVA) at a 0.05 significance level followed by least significant difference (LSD) tests. The virus variance between males and females was compared by Student’s *t*-test. All data analyses were performed using SAS 8.0.

## Results

### Efficiency of CLCuMuV and CLCuMuB transmission by whiteflies

Three species of whiteflies MEAM1, MED and Asia II 7 were compared for their efficiency of CLCuMuV and CLCuMuB transmission. Adult MEAM1 and MED whiteflies did not transmit CLCuMuV to 112-2, Xinhai-21 and Zhongmian-40, the transmission efficiency were 0 based on symptom inspection and PCR detection of viral DNA as well. Asia II 7 was able to transmit the virus, the average transmission efficiencies were 40.0%, 30.0% and 0 shown by both methods (Table 3). The symptoms appeared approximately 4–8 weeks after virus inoculation, included downward curling of leaf margins and vein swelling (Tang et al., 2015).

**Table 3:** Transmission efficiency of CLCuMuV to *Gossypium hirsutum* plants by Asia II 7, MEAM1, and MED whiteflies.

**Cryptic species**	**Variety**	**No. of tested plants**	**No. of whiteflies**	**No. of diseased plants**	**No. positive by PCR detection**	**Transmission efficiency (%)**
Asia II 7	112-2	20	10	8	8	40.00
	Xinhai-21	20	10	6	6	30.00
	Zhongmian-40	20	10	0	0	0
MEAM1	112-2	20	10	0	0	0
	Xinhai-21	20	10	0	0	0
	Zhongmian-40	20	10	0	0	0
MED	112-2	20	10	0	0	0
	Xinhai-21	20	10	0	0	0
	Zhongmian-40	20	10	0	0	0

### The accumulation of CLCuMuV in whitefly female and male adults

**Figure 1:**
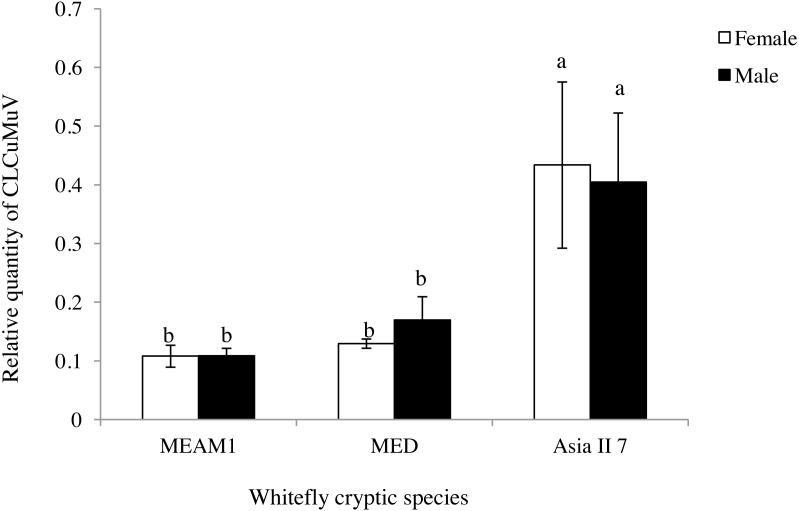
Relative quantity of CLCuMuV in the three cryptic whitefly species that fed on diseased *Gossypium hirsutum* plants with an acquisition access period of 48 h. Different letters in each figure represent significant differences at *P* < 0.05 (ANOVA: Duncan’s test). The same letters on columns represent no significant differences among one other when comparing the relative quantity of the virus in the same sexual form of the different cryptic species.

The difference in the quantity of CLCuMuV acquired between male and female adults that were fed CLCuMuV and CLCuMuB-infected cotton plants for 48 h was determined by qPCR. The quantity of virus in adult female or male insects of MEAM1, MED and Asia II 7 was not significantly different (*t* = 0.02, *P* = 0.9882; *t* =  − 1.04, *P* = 0.4006*; t* = 0.17*, P* = 0.8764), but the relative quantity of viral particles in Asia II 7 were significantly higher as compared to the rest of the two tested cryptic species (Fig. 1).This result indicated that the different sex of whitefly adults had no significant difference in ability to acquire the virus from CLCuMuV and CLCuMuB-infected source plants.

### Acquisition of CLCuMuV and CLCuMuB by whiteflies

**Figure 2:**
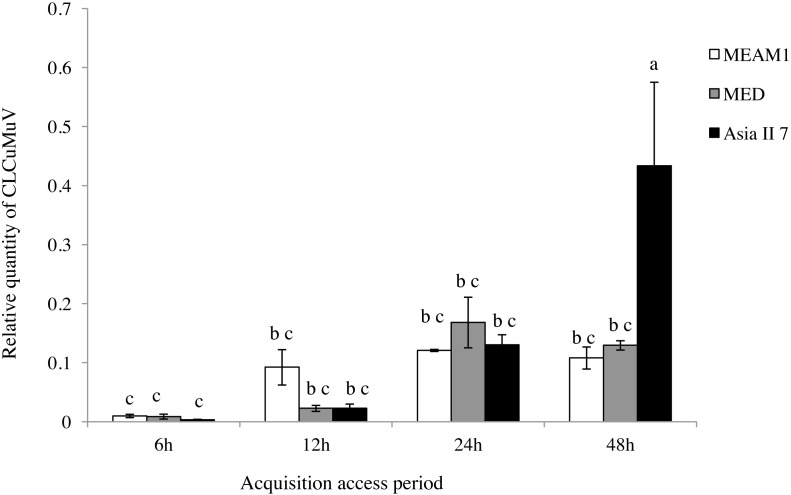
Relative quantity of CLCuMuV in three cryptic whitefly species quantified by qPCR. Columns with the same letter are not significantly different regarding the relative quantity of the virus in the same cryptic whitefly species fed on CLCuMuV and CLCuMuB- infected cotton for 6 h, 12 h, 24 h, and 48 h.

**Figure 3:**
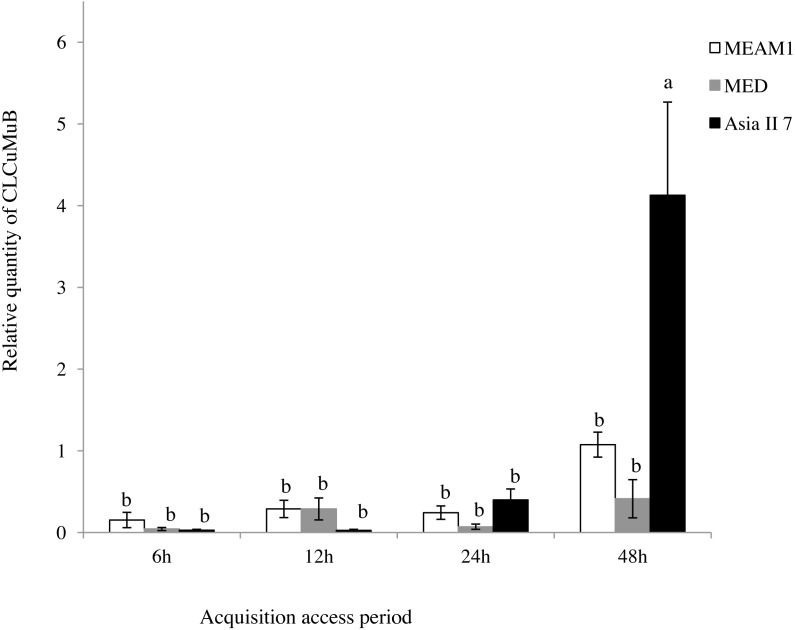
Relative quantity of CLCuMuB in three cryptic whitefly species quantified by qPCR. Different columns with the same letter are not significantly different regarding the relative quantity of the virus in the same cryptic whitefly species fed on CLCuMuV and CLCuMuB- infected cotton for 6 h, 12 h, 24 h, and 48 h.

A comparison of the relative quantity of virus in the three species of whitefly fed on CLCuMuV and CLCuMuB-infected cotton plants for a 6, 12, 24 and 48 h AAP was conducted. The results showed that all three species ingested both CLCuMuV and CLCuMuB. The relative quantity of CLCuMuV (Fig. 2) and CLCuMuB (Fig. 3) in the whiteflies increased significantly in each treatment i.e., 6 h, 12 h and 24 h of AAP (*F* = 1.45, *P* = 0.3061; *F* = 4.96, *P* = 0.0536; *F* = 0.77, *P* = 0.5050); however, the virus quantity after 48 h AAP in Asia II 7 was much higher than that in MEAM1 and MED (*F* = 4.09, *P* = 0.0212).

## Discussion

We have showed that all MEAM1, MED and Asia II 7 of *B. tabaci* tested in this study were able to acquire CLCuMuV and CLCuMuB, but only Asia II 7 was able to transmit CLCuMuV and CLCuMuB. The lower transmission capacity of MEAM1and MED is consistent with the results of Pan et al. (2018). It has been recognized that there are differences in transmission rates of begomoviruses owing to various whiteflies (Bedford et al., 1994; Jiu, Zhou & Liu, 2006; Wei et al., 2014). Evidence suggests that the distribution of CLCuD in South Asia is governed predominantly by a specific vector population, not by a host plant or geographic origin (Seal, Van Den Bosch & Jeger, 2006). Asia II 1 provided had the higher capacity than other species in the transmission of CLCuMuV among host plants of *Malvaceae* in China (Chen et al., 2016b; Pan et al., 2018). Even though Asia II 7 is not the predominant species in Pakistan and India (Islam et al., 2018; Ellango et al., 2015), while the cryptic species (Asia II 7) is found in China to be involved in CLCuMuV transmission. Based on Bayesian analysis of mtCO I haplotypes, Asia II 7 and Asia II 1 belong to the same Asia II genetic group (De Barro et al., 2010). It’s reasoned possibly that they have similarity in genomics and then share the capacity to transmit beogomovirus.

Begomoviruses are transmitted by whiteflies in a persistent and circulative way. Viruses acquisition, retention and transmission in host plants belonging to different families are associated with the successful spread of the viruses. The whitefly-mediated transmission of cotton leaf curl virus to cotton plants showed that the least amount of time required to acquire the virus by whiteflies was 15 min to 4 h, while the least amount of time required to inoculate the plant was from 5 min to 1 h (Mann & Singh, 2004). Based on qPCR analysis, all three *B. tabaci*-complex species acquired CLCuMuV and CLCuMuB after a 6 h, 12 h, 24 h and 48 h AAP. Whereas transmission experiments revealed that Asia II 7 could transmit the virus efficiently, but MEAM1 and MED were unable to transmit CLCuMuV and CLCuMuB, which might be due to a transmission barrier present in non-vector whiteflies. According to a previous report that employed immunofluorescence assays, CLCuMuV and CLCuMuB had a poorer capacity to cross the midguts of MEAM1 and MED than the midgut of Asia II 1 (Pan et al., 2018). Similarly, a study by Ohnishi et al. (2009) suggested that the greenhouse whitefly *Trialeurodes vaporariorum* could ingest and maintain *Tomato yellow leaf curl virus* after acquisition feeding on an infected plant; however, *T. vaporariorum* is a non-vector because of the presence of a selective transmission barrier at the luminal membrane surface of the epithelial cells of the midgut. The physiological differences in CLCuMuV and CLCuMuB transmission between Asia II 7 and the two invasive species need further study. Complete genomic information of Asia II 1 is studied (Hussain et al., 2019) and a similar study on Asia II 7 would be helpful to interpret the involvement of molecular factors in transmission capability of the newly identified vector in China.

In the present study, the transmission efficiency varied with the cotton variety. Asia II 7 was able to transmit CLCuMuV and CLCuMuB to the cotton varieties 112-2 and Xinhai-21, but not to Zhongmian-40. Furthermore, Asia II 7 transmitted the virus to variety 112-2 more efficiently than they did to Xinhai-21. Differences in transmission efficiency were assumed to be caused by cotton cultivars with different levels of resistance. Twenty-two cotton varieties were screened for resistance to CLCuD by graft inoculation in the laboratory and whitefly-vectored transmission assays in the field cages in Pakistan (Rahman et al., 2005), 14 varieties were susceptible and 8 resistant. They found three genes being involved in *G. hirsutum* resistance to CLCuD, two for resistance and a suppressor of resistance. Akhtar et al. (2008); Akhtar et al. (2010) tested cotton resistance to CLCuMuV and *cotton leaf curl Burewala virus* (CLCuBV) by graft inoculation, the results showed variation in the resistance of cotton varieties. Similar results were obtained when these cotton species were tested using whitefly-vectored transmission. Experimental inoculation of CLCuMuV and CLCuMuB infectious clones in 46 Chinese cotton varieties indicated that 4 varieties were highly susceptible to CLCuMuV (Tang et al., 2017). In our study, the three varieties come from different cotton cultivation research institute in China, which were differences in genetic backgrounds, so they were different in resistance to CLCuMuV. Host-plant resistance in cotton cultivars is the best long-term strategy to protect the crop against CLCuD (Jones, 2001). With the rapid spread and emergence of new begomoviruses throughout cotton growing areas of the world, further detailed research is required to be done to identify resistance in some cotton varieties against both vector and virus.

## Conclusions

Our results revealed that the native Asia II 7 was able to transmit CLCuMuV and CLCuMuB, the transmission ability of Asia II 7 varied with the cotton cultivars. We can quarantine and control whitefly vector and identify resistance in Gossypium species for slowing down the spread of CLCuMuV in China.

##  Supplemental Information

10.7717/peerj.7788/supp-1 Dataset S1 Efficiency of CLCuMuV transmission by whiteflies

10.7717/peerj.7788/supp-2 Dataset S2 The relative concentrations of CLCuMuV in adult whiteflies used for the transmission tests and were compared by one-way analysis of variance and T-test

## Additional Information and Declarations

### Data Availability

The following information was supplied regarding data availability: The raw measurements are available in the Supplemental Files.
