# Supplementary material for: Transmission efficiency of Cotton leaf curl Multan virus by three cryptic species of Bemisia tabaci complex in cotton cultivars
Source: PeerJ. 2019 Oct 1;7:e7788. doi: 10.7717/peerj.7788 (PMC6777476; doi:10.7717/peerj.7788)
Supplement: Dataset S1 [file peerj-07-7788-s001.docx]

Efficiency of CLCuMuV transmission by whiteflies.

| **Cryptic species** | Hostplant | No. of tested plants | No. of whiteflies | No. of diseased plants | Positive no. with PCR detection | Transmission efficiency |
| --- | --- | --- | --- | --- | --- | --- |
| Asia Ⅱ 7 | cotton112-2 | 20 | 10 | 7 | 7 | 35 |
|  | cotton112-2 | 20 | 10 | 8 | 8 | 40 |
|  | cotton112-2 | 20 | 10 | 8 | 8 | 40 |
|  | Xinhai-21 | 20 | 10 | 6 | 6 | 30 |
|  | Xinhai-21 | 20 | 10 | 6 | 6 | 30 |
|  | Xinhai-21 | 20 | 10 | 5 | 5 | 25 |
|  | Zhongmian | 20 | 10 | 0 | 0 | 0 |
|  | Zhongmian | 20 | 10 | 0 | 0 | 0 |
|  | Zhongmian | 20 | 10 | 0 | 0 | 0 |

| **Cryptic species** | Hostplant | No. of tested plants | No. of whiteflies | No. of diseased plants | Positive no. with PCR detection | Transmission efficiency |
| --- | --- | --- | --- | --- | --- | --- |
| **MEAM 1** | cotton112-2 | 20 | 10 | 0 | 0 | 0 |
|  | cotton112 | 20 | 10 | 0 | 0 | 0 |
|  | cotton112 | 20 | 10 | 0 | 0 | 0 |
|  | Xinhai-21 | 20 | 10 | 0 | 0 | 0 |
|  | Xinhai-21 | 20 | 10 | 0 | 0 | 0 |
|  | Xinhai-21 | 20 | 10 | 0 | 0 | 0 |
|  | Zhongmian-40 | 20 | 10 | 0 | 0 | 0 |
|  | Zhongmian-40 | 20 | 10 | 0 | 0 | 0 |
|  | Zhongmian-40 | 20 | 10 | 0 | 0 | 0 |

| **Cryptic species** | Hostplant | No. of tested plants | No. of whiteflies | No. of diseased plants | Positive no. with PCR detection | Transmission efficiency |
| --- | --- | --- | --- | --- | --- | --- |
| **MED** | cotton112-2 | 20 | 10 | 0 | 0 | 0 |
|  | cotton112 | 20 | 10 | 0 | 0 | 0 |
|  | cotton112 | 20 | 10 | 0 | 0 | 0 |
|  | Xinhai-21 | 20 | 10 | 0 | 0 | 0 |
|  | Xinhai-21 | 20 | 10 | 0 | 0 | 0 |
|  | Xinhai-21 | 20 | 10 | 0 | 0 | 0 |
|  | Zhongmian-40 | 20 | 10 | 0 | 0 | 0 |
|  | Zhongmian-40 | 20 | 10 | 0 | 0 | 0 |
|  | Zhongmian-40 | 20 | 10 | 0 | 0 | 0 |
