# Supplementary material for: Transmission efficiency of Cotton leaf curl Multan virus by three cryptic species of Bemisia tabaci complex in cotton cultivars
Source: PeerJ. 2019 Oct 1;7:e7788. doi: 10.7717/peerj.7788 (PMC6777476; doi:10.7717/peerj.7788)

The relative concentrations of CLCuMuV and betasatellite in adult whiteflies were used for the transmission tests and were compared by one-way analysis of variance (ANOVA) at a 0.05 significance level followed by least significant difference (LSD) tests.

MEAM1=B MED=Q Asia II 7=CV

MEAM1 48h T-test


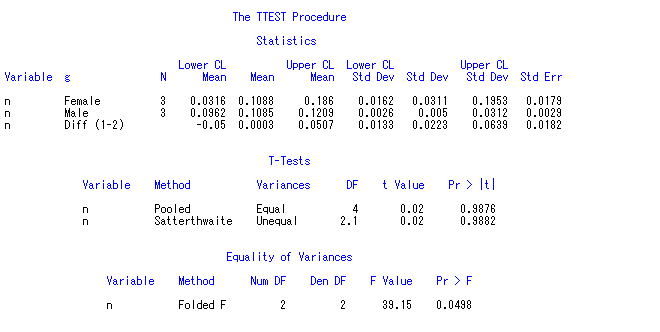


MED 48h T-test


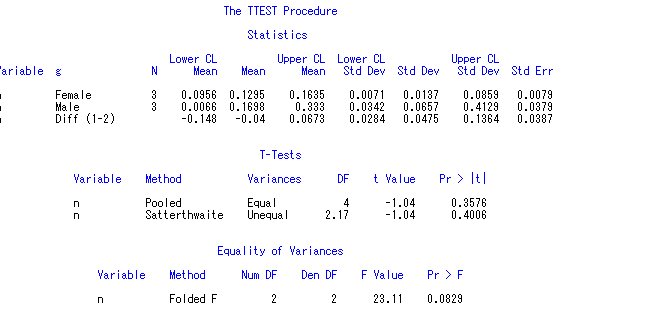


Asia II 7 48h T-test


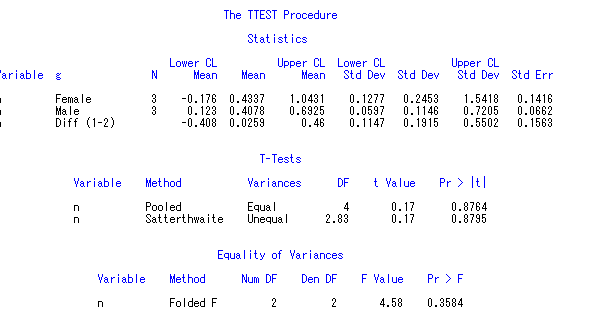


Relative concentrations of CLCuMuV in the three cryptic whitefly species that fed on diseased Gossypium hirsutum plants with an acquisition access period of 48 h.


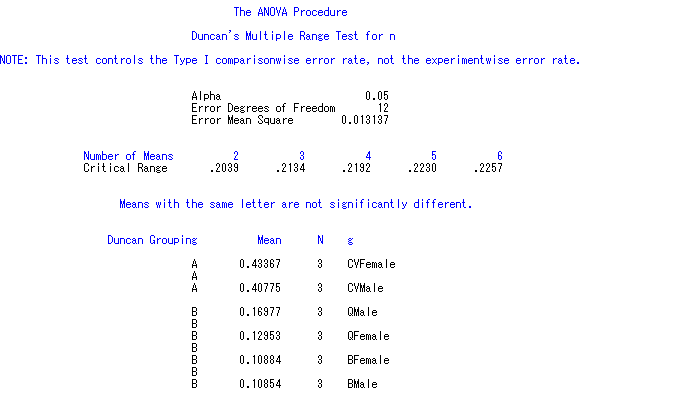


The relative concentrations of CLCuMuV


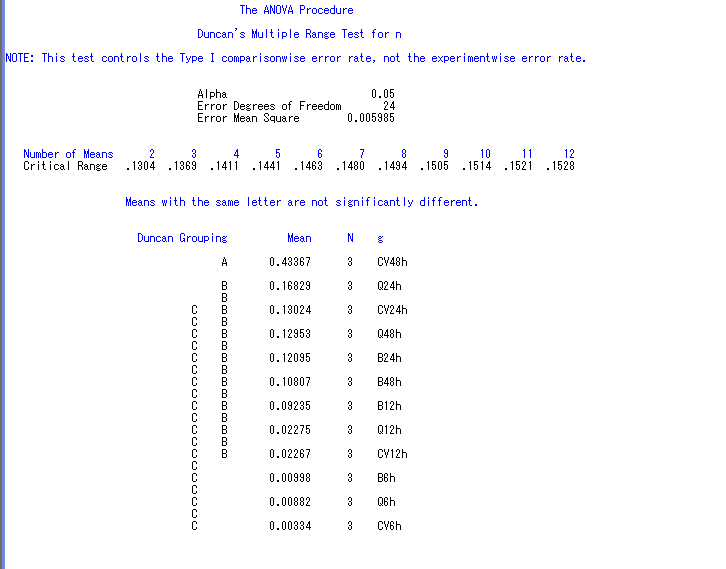


The relative concentrations of betasatellite


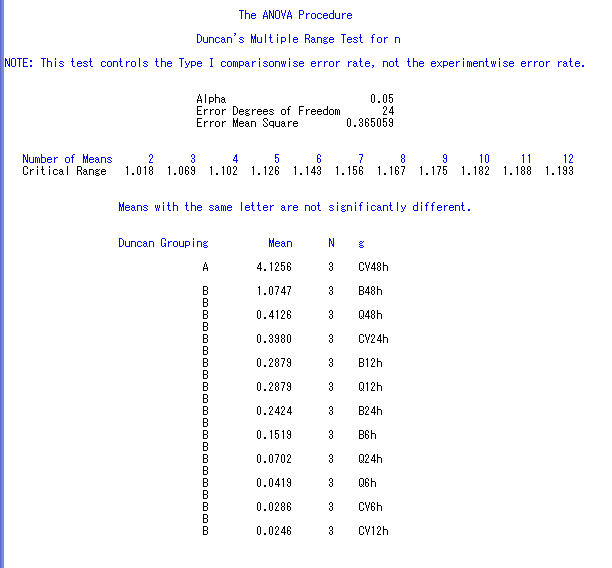

Supplement: Dataset S2 [file peerj-07-7788-s002.docx]
